# Interactive Associations between *PPARγ* and *PPARGC1A* and Bisphosphonate-Related Osteonecrosis of the Jaw in Patients with Osteoporosis

**DOI:** 10.3390/ph16071035

**Published:** 2023-07-21

**Authors:** Jung Sun Kim, Jin Woo Kim, Jeong Yee, Sun Jong Kim, Jee Eun Chung, Hye Sun Gwak

**Affiliations:** 1 Graduate School of Pharmaceutical Sciences, College of Pharmacy, Ewha Womans University, 52 Ewhayeodae-gil, Seodaemun-gu, Seoul 03760, Republic of Korea; junction717@ewhain.net (J.S.K.); jjjhello1@naver.com (J.Y.); 2 Department of Oral and Maxillofacial Surgery, School of Medicine, Ewha Womans University Medical Center, Mokdong Hospital, 1071 Anyangcheon-ro, Yangcheon-gu, Seoul 07985, Republic of Korea; jwkim84@ewha.ac.kr (J.W.K.); sjsj7777@ewha.ac.kr (S.J.K.); 3 Institute of Pharmaceutical Science and Technology, College of Pharmacy, Hanyang University, 55 Hanyangdaehak-ro, Sangnok-gu, Ansan 15588, Republic of Korea

**Keywords:** bisphosphonate-related osteonecrosis, *PPARγ*, *PPARGC1A*, gene polymorphism

## Abstract

Bisphosphonate-related osteonecrosis of the jaw (BRONJ) is a rare but severe adverse effect that can occur as a result of bisphosphonate treatment. This study aimed to examine the relationship between *PPARγ* and *PPARGC1A* polymorphisms and the BRONJ development in female osteoporosis patients undergoing bisphosphonate treatment. We prospectively conducted this nested case–control study at the Ewha Womans University Mokdong Hospital between 2014 and 2018. We assessed five single-nucleotide polymorphisms (SNPs) of *PPARγ* and six SNPs of *PPARGC1A* and performed a multivariable logistic regression analysis to determine the independent risk factors for developing BRONJ. There were a total of 123 patients included in this study and 56 patients (45.5%) developed BRONJ. In the univariate analysis, *PPARGC1A* rs2946385 and rs10020457 polymorphisms were significantly associated with BRONJ (*p* = 0.034, *p* = 0.020, respectively), although the results were not statistically significant in the multivariable analysis. Patients with the combined genotypes of GG in both *PPARγ* rs1151999 and *PPARGC1A* rs2946385 showed a 3.03-fold higher risk of BRONJ compared to individuals with other genotype combinations after adjusting for confounders (95% confidence interval (CI): 1.01–9.11). Old age (≥70 years) and duration of bisphosphonate use (≥60 months) increased the risk of BRONJ. The area under the receiver operating characteristic curve for the predicted probability was 0.78 (95% CI: 0.69–0.87, *p* < 0.001), demonstrating a satisfactory level of discriminatory power. Our study elucidated that *PPARγ* and *PPARGC1A* polymorphisms were interactively associated with BRONJ development. These results have potential implications for tailoring personalized treatments for females undergoing bisphosphonate therapy for osteoporosis.

## 1. Introduction

Bisphosphonates are widely used to treat bone diseases, such as osteoporosis and cancer-induced bone metastasis. They impair osteoclast activity, thereby increasing bone mass [1]. While bisphosphonates are generally well tolerated, they can cause renal toxicity, gastric ulcers, and osteonecrosis of the jaw (ONJ) [2].

Bisphosphonate-related ONJ (BRONJ) is a rare but severe adverse effect related to bisphosphonate therapy [3]. Clinical BRONJ manifestations include the presence of exposed bone in the maxillofacial area that persists for more than eight weeks in patients who have been administered bisphosphonates, particularly without any prior history of radiation therapy [4]. Since the first case reported by Marx in 2003, many studies have been released to distinguish the pathophysiology and risk factors of BRONJ [5]. Nevertheless, the precise underlying cause remains unidentified. Some intriguing pathogenic mechanisms have been proposed, such as the disturbance of the normal bone turnover cycle, the hindrance of the cellular wound healing process, and impaired angiogenesis [6,7,8]. In terms of risk factors, several have been reported in association with BRONJ development, including underlying malignant disease, the intravenous use of bisphosphonates, prolonged or high-dose bisphosphonate therapy, concomitant use of certain medications, dental infections, and jawbone surgical procedures [9]. In addition to environmental factors, genetic predisposition has been identified as a risk factor [10,11]. Several studies have reported BRONJ-associated genes such as *CYP2C8*, *RBMS3*, *SIRT1*, *COL1A1*, *RANK*, *CYP19A1*, *VEGFA*, and *PPARγ* through genome-centered or single-nucleotide polymorphism (SNP) analyses [12,13,14,15,16].

Peroxisome proliferator-activated receptor gamma (PPARγ), a nuclear receptor of transcription factors, regulates various physiological processes, such as adipogenesis, glucose and lipid metabolism, inflammation, angiogenesis, and bone remodeling [17,18,19,20]. In bone remodeling, PPARγ inhibits the differentiation of mesenchymal stem cells into osteoblasts and promotes the differentiation of hematopoietic stem cells into osteoclasts. PPARγ modulates transcription by recruiting coactivators [18]. One specific coactivator of PPARγ in bone homeostasis is PPARγ coactivator 1 alpha (PPARGC1A). Recent studies have reported the associations between *PPARGC1A* and the regulation of bone homeostasis [21,22]. Regarding these findings, genetic variants of *PPARγ* and *PPARGC1A* may affect bisphosphonate’s mechanism of action in bone remodeling, contributing to the development of BRONJ.

The association between *PPARγ* polymorphisms and the onset of BRONJ has been demonstrated in previous research [16,23,24]. However, a meta-analysis found no significant relationship between this gene and the BRONJ with low-quality evidence [10]. The majority of studies concentrated on patients with multiple myeloma or solid cancers, leaving a research gap regarding osteoporosis patients. Moreover, the impact of *PPARGC1A* polymorphisms on BRONJ has not been investigated. Our study objective was to examine the relationship between *PPARγ* and *PPARGC1A* polymorphisms and the development of BRONJ in patients with osteoporosis patients undergoing bisphosphonate treatment.

## 2. Results

Of the 147 patients who were initially screened, 24 were excluded as 2 were male, 20 used bisphosphonates for cancer, and 2 had insufficient clinical data. Consequently, a total of 123 patients were eligible for this study, among whom 56 patients (45.5%) developed BRONJ. The mean age and duration of bisphosphonate use were 72.9 ± 9.5 years and 54.7 ± 79.1 months, respectively. The relationship between baseline characteristics and BRONJ is demonstrated in Table 1. Age was statistically significantly associated with BRONJ, as individuals aged over 70 years were at a higher risk compared to others (*p* = 0.003). The BRONJ group had a higher hypertension prevalence compared to the non-BRONJ group (62.5% vs. 41.8%, *p* = 0.022). No significant differences were observed in the types and routes of bisphosphonate administration between the cases and controls. However, there was a trend towards an increased risk of BRONJ with a bisphosphonate use duration of 60 months or longer (*p* = 0.001).

Table 2 displays the genotypes and minor allele frequency (MAF) of the analyzed SNPs in *PPARγ* and *PPARGC1A*. Among the *PPARγ* SNPs studied, there were no significant SNPs showing an association with BRONJ. However, when examining *PPARGC1A*, rs2946385 and rs10020457 were significantly associated with the occurrence of BRONJ. Additionally, when considering the combination of *PPARγ*/*PPARGC1A* SNPs, rs1151999/rs294638 and rs1151999/rs10020457 displayed a significant association with BRONJ.

As shown in Table 3, in the univariate analysis, patients with the GG genotype of *PPARGC1A* rs2946385 had a higher frequency of BRONJ development than individuals with the T allele (Odds ratio (OR) 2.18; 95% confidence interval (CI): 1.06–4.50). For *PPARGC1A* rs10020457, individuals with the A allele had a 3.0-fold increased risk of developing BRONJ compared to those with GG genotypes (95% CI: 1.15–7.54). We conducted the multivariable analyses using three models to evaluate the independent and interactive associations between the SNPs and BRONJ. Model I included demographic and genetic factors that exhibited a *p* < 0.1 in the univariate analysis. Only age ≥70 years and a duration of bisphosphonate use ≥60 months were statistically significantly associated with BRONJ after adjusting for the covariates. 

**Figure 1:**
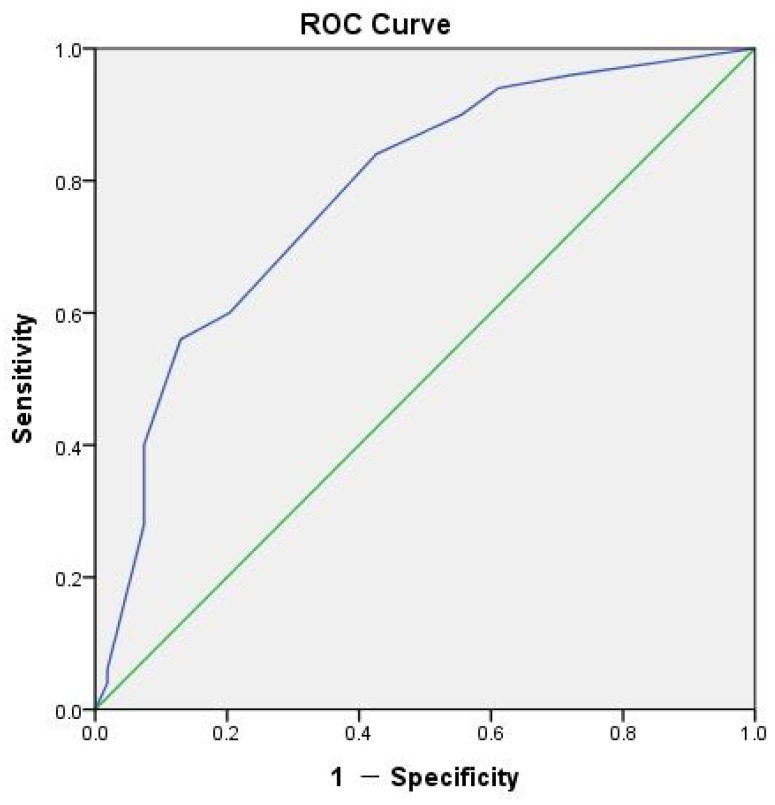
The area under the receiver operating curve (ROC) for the development of bisphosphonate-related osteonecrosis of the jaw. The blue line presents the predicted probability of Model II (demographic factors and the combination of *PPARγ* rs1151999 GG and *PPARGC1A* rs2946385 GG genotypes), while the green line presents the reference.

To assess the interactive effect of combined genotypes, we constructed additional models. Model II involved the combination of *PPARγ* rs1151999 GG and *PPARGC1A* rs2946385 GG genotypes, while Model III involved *PPARγ* rs1151999 GT and *PPARGC1A* rs10020457 GA genotypes. In Model II, patients with the combined genotypes of GG in both *PPARγ* rs1151999 and *PPARGC1A* rs2946385 showed a higher risk of developing BRONJ compared to patients with other genotype combinations (adjusted odds ratio (AOR): 3.03, 95% CI: 1.01–9.11). However, the effect of combined genotypes in Model III was not significant in the multivariable analysis. Regardless of the models, age (≥70 years) and duration of bisphosphonate use (≥60 months) remained significant factors. The result of the Hosmer–Lemeshow test of Model II indicated a reasonably good fit to the observed data (χ^2^ = 3.998, 7 degrees of freedom, and *p* = 0.780). The area under the receiver operating curve (AUROC) for the predicted probability of the multivariable logistic regression for Model II was 0.78 (95% CI: 0.69–0.87, *p* < 0.001; Figure 1), demonstrating a satisfactory level of discriminatory power.

## 3. Discussion

Previous studies have demonstrated the relationship between *PPARγ* and BRONJ; however, conflicting results were found. Furthermore, these studies were confined to cancer patients, leaving a research gap concerning patients with osteoporosis. In this study, our objective was to investigate the association between *PPARγ* and *PPARGC1A* polymorphisms and the development of BRONJ in osteoporosis patients undergoing bisphosphonate treatment. This study indicated that the combined effect of *PPARγ* rs1151999 GG and *PPARGC1A* rs2946385 GG genotypes was related to BRONJ, along with age and treatment duration after adjusting for confounders. The AUROC for predicted probability revealed good model performance.

Although the individual SNPs of *PPARγ* rs1151999 and *PPARGC1A* rs2946385 were not statistically significant in the multivariable analysis, a significant interactive effect was observed when these SNPs were analyzed in combination. PPARGC1A, a coactivator of PPARγ, interacts with factors involved in RNA splicing and transcript elongation, suggesting a potential role in mRNA maturation [25]. A study conducted by Ruchat et al. reported independent and interactive associations between *PPARγ* and *PPARGC1A* with insulin and glucose homeostasis [26]. Subjects who had the reference allele in *PPARGC1A* and the variant allele of *PPARγ* exhibited higher fasting insulin levels, insulin resistance, and insulin area under the curve compared to those with the other combinations. An interactive association could occur when both the transcriptional factor and its coactivator possess the polymorphism, resulting in maximal and synergistic effects on the development of BRONJ.

PPARγ, located on chromosome 3p25.3, plays a vital role in maintaining skeletal homeostasis. Prolonged use of thiazolidinediones, which are synthetic agents that activate PPARγ, has been correlated with an increased incidence of fracture in individuals with diabetes mellitus [27,28]. The study findings revealed that patients with osteoporosis exhibited an increased number of adipose cells in the bone marrow. This was observed as a result of the stimulation of PPARγ, which led to the differentiation of the stem cells into adipocytes. The association between increased adipose cells and osteoporosis can be attributed to the inhibitory effect of PPARγ on bone formation [29,30]. Hence, polymorphisms in the *PPARγ* gene are often linked to a higher susceptibility to skeletal disorders. A cohort study conducted in Denmark found that genetic variation increased the risk of vertebral fracture [31]. A pilot study conducted by Dragojevič et al. also indicated that the *PPARγ* polymorphism could play a role in the incidence of non-traumatic hip fractures among older individuals [32].

Regarding BRONJ, several studies have been conducted on the *PPARγ* rs1152003 polymorphism. However, the recently published meta-study did not find a significant association between the polymorphism of rs1152003 and the incidence of BRONJ [10]. Similarly, our study observed no significant difference in the association of *PPARγ* rs1152003 with BRONJ. With respect to *PPARγ* rs1151999, it did not show a significant association with BRONJ, whereas its combination with either *PPARGC1A* rs2946385 or rs10020457 demonstrated a significant correlation with BRONJ in the univariate analysis. Although rs1151999 is an intron variant, intron regions may impact mRNA splicing, thereby modifying protein expression or activity [33,34]. Kiel et al. showed that a haplotype containing the G allele of rs1151999 was associated with decreased femoral neck bone mineral density [35]. This finding is consistent with our study, which showed that the GG genotype was associated with an increased risk of BRONJ, likely due to decreased bone mineral density. In addition to bone-related effects, the polymorphism of this gene has been shown to impact other systems significantly. Cirelli et al. demonstrated that the wild-type allele of rs1151999 significantly reduced the risk of hyperglycemia, hyperlipidemia, and obesity even after adjustments for age, sex, and smoking status in a Brazilian population [36]. Moreover, the rs1151999 SNP was associated with protection against Alzheimer’s disease [37]. While this potential mechanism is intriguing, there is no evidence that rs1151999 directly affects *PPARγ* mRNA expression levels. Further research is needed for a more comprehensive understanding of this SNP.

PPARGC1A is a transcriptional coactivator that interacts with PPARγ, allowing it to interact with multiple transcription factors. While the role of PPARGC1A in energy metabolism is well established, its impact on bone in humans has been barely studied. However, recent in vitro and animal studies have shown the involvement of PPARGC1A in bone-related processes. In in vitro studies, it positively influenced SIRT3 activity, thereby promoting osteogenic differentiation [38] and increasing the expression of essential genes related to osteocyte function [39]. In aged mice, a *PPARGC1A* deficiency has negative effects on bone mass and strength [40]. *PPARGC1A*-deficient mice exhibited impaired bone structure and lower trabecular thickness, leading to a significant decline in bending strength. Consistently, Yu et al. demonstrated that *PPARGC1A* regulated the balance between bone and fat in a mouse model of osteoporosis. Loss of function of this gene in skeletal stem cells inhibited bone formation and facilitated the accumulation of adipose tissue in the bone marrow, while the induction alleviated bone loss [21].

PPARGC1A also plays a crucial role in the angiogenesis and migration of associated endothelial cells [41]. The growth of blood vessels is crucial for initiating and sustaining the wound healing process. Bi et al. demonstrated that bisphosphonate was the primary cause of BRONJ-like disease in mice, mediated in part by its ability to suppress osseous angiogenesis [42]. Consequently, the anti-angiogenic effect has been considered as a possible mechanism underlying BRONJ in humans. Arduino et al. investigated the polymorphism of the vascular endothelial growth factor (*VEGF*) gene in relation to female patients who developed BRONJ in Italy, and the results suggested a possible haplotype effect of *VEGF* polymorphism expression [15]. In a Korean cohort, *VEGF* polymorphisms showed a significant association with an increased risk of BRONJ [43]. These findings imply that the *PPARGC1A* polymorphisms can contribute to the pathogenesis of BRONJ through multiple mechanisms.

*PPARGC1A* rs2946385, a synonymous variant, was a significant risk factor in our study. While the specific function of this SNP in maintaining bone homeostasis has not been thoroughly characterized, it has been investigated in relation to type 2 diabetes mellitus (T2DM). A study conducted on Indian patients indicated that the variant allele was associated with increased susceptibility to T2DM (OR 1.72, 95% CI: 1.05–2.82) [44]. This finding could be interpreted that the polymorphism resulted in decreased mRNA expression of PPARGC1A, which eventually influenced insulin secretion. In contrast, this SNP polymorphism did not demonstrate any significant association with T2DM in the Caucasian population [45]. The inconsistent findings could be attributed to ethnic diversity or possible interactions among genetic and environmental factors.

Among environmental factors, age and the duration of bisphosphate use are widely acknowledged as potential risk factors associated with BRONJ [9,46]. In a large population-based study involving 58,069 participants, individuals who were 65 years or older exhibited a 3.0-fold (95% CI: 2.1–4.2) higher risk of BRONJ compared to those in the age group of 20–64 years [47]. In our study, hypertension was associated with a 2.1-fold higher risk of BRONJ, although statistical significance was not found in the multivariable analysis. There is no specific report providing detailed information on the relationship between these two factors, but this association could be explained by the higher prevalence of hypertension among older patients.

Another identified risk factor for BRONJ is the duration of bisphosphonate use. The onset of BRONJ differs depending on the potency of bisphosphonates as intravenous administration results in higher drug exposure compared to oral administration [48]. A prospective study investigating intravenous bisphosphonate (pamidronate and zoledronate) demonstrated that bisphosphonate use over 30 months increased the risk of developing ONJ [49]. However, a review of 103 patients who received oral bisphosphonates (alendronate and risedronate) reported a mean time to the onset of the disease as 4.6 years [50]. In our study, 74 (60.2%) patients were administered bisphosphonate orally, and 12 (9.8%) patients who initially received bisphosphonates intravenously switched to the oral route. Consistent with these findings, our study revealed that advanced age and long-term use of bisphosphonate were associated with a higher risk of BRONJ.

Some limitations need to be addressed. First, the sample size is relatively small. Second, potential confounding factors, such as smoking history and medication use, were not considered in the analysis, which could affect the occurrence of BRONJ. Third, the exclusion of two male patients to focus on gender-specific effects may limit the generalizability of the findings. Lastly, we could not measure the sole impact of the administration route because many patients who initially received bisphosphonates intravenously switched to the oral route. Additional comprehensive cohort studies are necessary to account for potential confounders and validate our findings.

## 4. Materials and Methods

### 4.1. Study Participants and Data Collection

We prospectively conducted this nested case–control study at the Ewha Womans University Mokdong Hospital between January 2014 and December 2018. Among the patients scheduled for dentoalveolar surgery, those who were currently or previously taking bisphosphonates were enrolled in this study. The inclusion criteria were patients aged 50 years or older with confirmed osteoporosis by a medical professional. Patients were excluded if they met any of the following criteria: (i) male sex, (ii) had cancer requiring antiresorptive drug treatment, or (iii) had insufficient clinical data or sample for DNA analysis. The diagnosis of BRONJ followed the guidelines outlined by the American Association of Oral and Maxillofacial Surgeons [48] and was made by maxillofacial surgeons. According to the guidelines, cases were considered to have developed BRONJ if all the following clinical manifestations were present: (i) the patient was receiving bisphosphonate treatment when BRONJ occurred, (ii) the exposed and necrotic bone in the maxillofacial area persisted for more than 8 weeks, and (iii) the patient had no history of head or neck radiation therapy. Controls consisted of patients under bisphosphonate treatment without any signs of BRONJ.

The study protocol was reviewed and approved by the Institutional Review Board (IRB) of Ewha Womans University Mokdong Hospital (IRB Number: 14-13-01). This study followed the ethical principles outlined in the 1694 Declaration of Helsinki and its subsequent amendments. We obtained approval from the ethics committee to ensure adherence to the required ethical standards for human research. All participants provided written informed consent before enrollment.

We reviewed the electronic medical records, gathering information such as age, gender, co-morbidities, the indication of bisphosphonates, types and doses of bisphosphonates, bisphosphonate therapy duration, and administration method.

### 4.2. Selection of Single-Nucleotide Polymorphisms (SNPs) and Genotyping Methods

We referenced previous research to select SNPs and determine the genotyping methods [31,51,52,53,54,55]. Based on this information, we identified five SNPs of *PPARγ* (one missense SNP (rs1801282), one synonymous SNP (rs3856806), one 3′-untranslated region (UTR) SNP (rs1152003), and two intron SNPs (rs1151999 and rs1175543)) and six SNPs of *PPARGC1A* (two missense SNPs (rs8192678 and rs3736265), two synonymous SNPs (rs2946385 and rs2970847), and two intron SNPs (rs10020457 and rs7665116). We obtained the details regarding these 11 SNPs, including chromosomal position, reference/alternate alleles, and functional information, from the SNP database of the National Center for Biotechnology Information. We collected Asian MAF and linkage disequilibrium (LD) data from Haploreg v4.2 [56]. 

Saliva samples were collected for genomic DNA using the tube format (OG300) of the Oragene^®^ DNA Self-Collection Kit (DNAgenotek, Kanata, ON, Canada). The selected 11 SNPs were genotyped using the TaqMan assay. The RT-PCR was performed using the TaqMan^®^ allele discrimination technique on an ABI 7300 instrument (Applied Biosystems, Carlsbad, CA, USA). The PCR was conducted in a 25 μL optical 8-cap strip, consisting of 11.25 μL of DNA samples and 13.75 μL of PCR mix. The PCR reagent mixture included 12.5 μL of the TaqMan Genotyping Master Mix and 1.25 μL of the 20× TaqMan SNP Genotyping Assay Mix (Applied Biosystems in Foster City, CA, USA). Following a denaturation step at 95 °C for 10 min, the PCR was carried out for 40 cycles at 92 °C for 15 s and 60 °C for 60 s. 

### 4.3. Statistical Analysis

We used the chi-squared test and Fisher’s exact test to compare categorical variables between patients with and without BRONJ. We analyzed each SNP in both dominant and recessive models and selected the appropriate model considering the effect size and statistical significance. As PPARGC1A is a coactivator of PPARγ, we also investigated the combined genetic effects of *PPARγ*/*PPARGC1A* on BRONJ development by grouping genotypes with similar trends. 

To determine the independent risk factors for BRONJ, we conducted a multivariable regression analysis, including variables with a *p* < 0.1 from the univariate analysis. We entered the variables by stepwise selection when the *p*-value was lower than 0.05 and removed them when it was higher than 0.1. The unadjusted OR and AOR with the 95% CI were calculated from univariate and multivariable analyses, respectively. The Hosmer–Lemeshow goodness-of-fit test was utilized to evaluate the adequacy of the prediction model’s fit. We evaluated the model’s discriminatory power using the AUROC analysis. A value of *p* < 0.05 indicated statistical significance. We used SPSS version 20 (IBM, Chicago, IL, USA) for all statistical analyses. 

## 5. Conclusions

Our findings demonstrated an interactive association between the polymorphisms in *PPARγ* rs1151999/*PPARGC1A* rs2946385 with the development of BRONJ, along with age (≥70 years) and duration of bisphosphonate use (≥60 months). After validation in a larger cohort study, these results have the potential to facilitate early identification of high-risk patients and inform personalized osteoporosis treatment strategies for individuals undergoing bisphosphonate therapy. 

## Figures and Tables

**Table 1:** Demographic characteristics of study participants.

Characteristics	Case (*n* = 56)	Control (*n* = 67)	*p*-Value
Age (years)			0.003
<70	9 (16.1)	27 (40.9)	
≥70	47 (83.9)	39 (59.1)	
Co-morbidity			
Hypertension	35 (62.5)	28 (41.8)	0.022
Cardiovascular disease	7 (12.5)	8 (11.9)	0.925
Diabetes mellitus	17 (30.4)	16 (23.9)	0.419
Rheumatoid arthritis	6 (10.7)	2 (3.0)	0.140 ^a^
Thyroid disease	4 (7.1)	2 (3.0)	0.286
Kidney disease	2 (3.6)	3 (4.5)	1.000 ^a^
Liver disease	0 (0)	2 (3.0)	0.500 ^a^
Cancer	2 (3.6)	6 (9.1)	0.289 ^a^
Types of bisphosphonates *			0.225 ^a^
Alendronate	9 (34.6)	12 (36.4)	
Ibandronate	5 (19.2)	12 (36.4)	
Pamidronate	2 (7.7)	0 (0)	
Risedronate	10 (38.5)	9 (27.3)	
Route of administration			0.210
Intravenous	9 (20.5)	18 (31.6)	
Oral	35 (79.5)	39 (68.4)	
Treatment duration (months)			0.001
<60	26 (52.0)	45 (83.3)	
≥60	24 (48.0)	9 (16.7)	

^a^ Fisher’s exact test, * including alendronate, ibandronate, pamidronate, and risedronate.

**Table 2:** Association of *PPARγ* and *PPARGC1A* gene polymorphisms with bisphosphonate-related osteonecrosis of the jaw.

Gene Polymorphism	MAF	Grouped Genotypes	Case (*n* = 56)	Control (*n* = 67)	*p*-Value
*PPARγ*					
rs1151999	0.390	GG, GT	52 (92.9)	55 (82.1)	0.077
G > T		TT	4 (7.1)	12 (17.9)	
rs3856806	0.107	CC	46 (82.1)	53 (80.3)	0.796
C > T		CT, TT	10 (17.9)	13 (19.7)	
rs1152003	0.402	GG, GC	47 (83.9)	50 (74.6)	0.208
G > C		CC	9 (16.1)	17 (25.4)	
rs1801282	0.041	CC	53 (94.6)	61 (91.0)	0.508 ^a^
C > G		CG, GG	3 (5.4)	6 (9.0)	
rs1175543	0.402	AA, AG	50 (90.9)	55 (82.1)	0.162
A > G		GG	5 (9.1)	12 (17.9)	
*PPARGC1A*					
rs8192678	0.415	CC	22 (39.3)	19 (28.4)	0.200
C > T		CT, TT	34 (60.7)	48 (71.6)	
rs2946385	0.291	GG	35 (62.5)	29 (43.3)	0.034
G > T		GT, TT	21 (37.5)	38 (56.7)	
rs10020457	0.102	GG	40 (71.4)	59 (88.1)	0.020
G > A		GA, AA	16 (28.6)	8 (11.9)	
rs7665116	0.260	TT	27 (48.2)	41 (61.2)	0.149
T > C		TC, CC	29 (51.8)	26 (38.8)	
rs2970847	0.240	TT, TC	23 (41.1)	29 (43.3)	0.805
T * > C		CC	33 (58.9)	38 (56.7)	
rs3736265	0.146	GG	38 (67.9)	53 (79.1)	0.157
G > A		GA, AA	18 (32.1)	14 (20.9)	
*PPARγ*/*PPARGC1A*					
rs1151999/rs2946385	N/A	GG/GG	16 (28.6)	9 (13.4)	0.038
		Others	40 (71.4)	58 (86.6)	
rs1151999/rs10020457	N/A	GT/GA	12 (21.4)	2 (3.0)	0.001
		Others	44 (78.6)	65 (97.0)	

^a^ Fisher’s exact test; * minor allele; MAF: minor allele frequency.

**Table 3:** Multivariable regression analyses to identify predictors of bisphosphonate-related osteonecrosis of the jaw.

Variables	Crude OR (95% CI)	Adjusted OR (95% CI)		
		Model I	Model II	Model III
Age ≥ 70 years	3.62 (1.52–8.59) **	3.94 (1.50–10.30) **	3.23 (1.18–8.84) *	4.27 (1.57–11.64) **
Hypertension	2.32 (1.12–4.80) *		2.13 (0.87–5.24)	
Duration ≥ 60 months	4.62 (1.87–11.42) ***	3.32 (1.28–8.59) *	4.27 (1.55–11.77) **	3.07 (1.17–8.07) *
*PPARγ* rs1151999 GG, GT	2.83 (0.86–9.09)			
*PPARGC1A* rs2946385 GG	2.18 (1.06–4.50) *			
*PPARGC1A* rs10020457 GA, AA	2.95 (1.15–7.54) *			
*PPARγ*/*PPARGC1A* rs1151999/rs2946385 GG/GG	2.41 (1.17–4.99) *		3.03 (1.01–9.11) *	
*PPARγ*/*PPARGC1A* rs1151999/rs10020457 GT/GA	3.43 (1.29–9.08) *			5.12 (0.89–29.57)

Model I included all factors with *p* < 0.1 in univariate analysis. Model II and Model III included combined genotype of *PPARγ*/*PPARGC1A* rs1151999/rs2946385 and *PPARγ*/*PPARGC1A* rs1151999/rs10020457 instead of individual SNPs in Model I, respectively. OR: odds ratio, CI: confidence interval. * *p* < 0.05, ** *p* <0.01, *** *p* < 0.001.

## Data Availability

Data is contained within the article.
